# Edge effect influences the ecological strategies of plant communities in tropical forest fragments

**DOI:** 10.1111/plb.70137

**Published:** 2025-11-11

**Authors:** T. S. Sousa, R. D. Pacheco, L. Pereira, A. Barbosa, L. G. Botelho, T. S. Michelan, R. M. Cerqueira, E. S. C. Gurgel, G. S. Teodoro

**Affiliations:** ^1^ Programa de Pós‐Graduação em Ecologia Universidade Federal do Pará Belém Brazil; ^2^ Ulm University Ulm Baden‐Wurttemberg Germany; ^3^ Programa de Pós‐Graduação em Botânica Tropical Museu Paraense Emílio Goeldi and Universidade Federal Rural da Amazônia Belém Brazil; ^4^ Programa de Pós‐Graduação em Ecologia Aplicada Universidade Federal de Lavras Lavras Brazil; ^5^ Instituto de Ciências Biológicas Universidade Federal do Pará Belém Brazil

**Keywords:** ecological strategies, edge effects, forest fragmentation, functional traits, wood–leaf decoupling

## Abstract

- The continuous fragmentation of tropical forests is a major threat to biodiversity and ecosystem functioning. This process creates extensive forest edges, alters microclimates, and promotes shifts in species composition. Functional traits are key to understanding how species respond to these disturbances and to predicting future vegetation dynamics. This study investigates the ecological strategies of species located at the edges and interiors of forest fragments in the Eastern Amazon.
- We sampled abundant tree species in seven forest fragments distributed across three municipalities in Pará, Brazil. We analysed 16 morphological and anatomical traits related to leaf economics and xylem function. Comparisons were made between edge and interior environments, and traits were correlated with edaphic variables.
- Species at forest edges had traits associated with hydraulic efficiency, including higher hydraulic conductivity and a greater fiber fraction. In contrast, interior species displayed a range of strategies, from resource‐acquisitive to conservative. We found evidence of a decoupling between leaf and wood trait axes, with wood traits varying independently from leaf traits. Soil conditions influenced trait patterns only at fragment edges.
- Our study enhances understanding of the mechanisms regulating species survival, as evidenced by the different strategies adopted by plants in the interior and at the edges of forest fragments, reflecting contrasting responses to resource availability. These findings also provide support for conservation and forest management strategies and contribute to policy development aimed at mitigating the impacts of fragmentation on Amazonian biodiversity.

## Graphical abstract

The edge effect in Eastern Amazonia influences ecological strategies of plant communities, with forest edges promoting traits linked to hydraulic efficiency, while interior species exhibiting diverse ecological strategies.

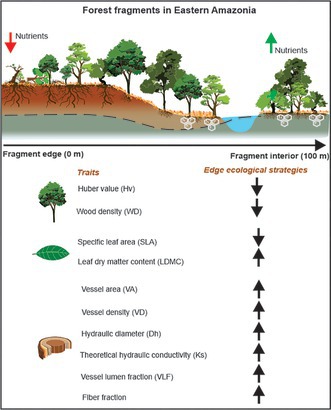

## INTRODUCTION

The continuing fragmentation of tropical forests is one of the greatest threats to global biodiversity (Laurance *et al*. 2006a; Broadbent *et al*. 2008). This process results in the rapid loss of habitat, an increase in the extent of forest edges, and the subdivision of large continuous areas into smaller, less diverse and isolated fragments (Bauer *et al*. 2024). Consequently, the edge effect alters the microclimate in these regions, increasing temperatures and light incidence, reducing humidity, and intensifying winds (Laurance *et al*. 2011; Qie *et al*. 2017; Laurance 2000; Lapola *et al*. 2023). These changes may promote the replacement of late‐successional trees by fast‐growing, light‐wooded pioneer species, although edges also harbour some late‐successional species (Laurance *et al*. 2002), which directly impacts the carbon cycle (Brinck *et al*. 2017). Thus, forest fragments harbour both pre‐existing trees in the interior and those that colonized the edges following the disturbance (Maeda *et al*. 2022; Nunes *et al*. 2023). However, we still have limited understanding of which functional traits are being maintained in the abundant species in forest fragments.

Functional traits are essential tools for characterizing the ecological strategies of plants and understanding their responses to fragmentation (Violle *et al*. 2007; Reich 2014). These functional traits reflect the morphological, physiological, and phenological adaptations that influence species' growth, reproduction, and survival (Violle *et al*. 2007; Reich 2014). In the context of forest fragmentation, it is essential to understand the ecological strategies of the species that persist in these forest fragments (Pinho *et al*. 2025). The leaf economic spectrum (Wright *et al*. 2004) and the wood economic spectrum (Chave *et al*. 2009) describe a continuum axis of plant functional strategies in response to resource availability and environmental pressures (Botelho *et al*. 2025). Species with more acquisitive strategies have high specific leaf area (SLA), low leaf dry matter content (LDMC), and lower wood density (WD), maximizing rapid growth in favourable environments. In contrast, species with conservative strategies display opposite patterns, favouring longevity and resilience under conditions of resource scarcity (Wright *et al*. 2004; Chave *et al*. 2009; Reich 2014).

Stem traits also play a fundamental role in species' response to environmental conditions and in regulating water transport (Apgaua *et al*. 2015). Wood density (WD) and the sapwood‐to‐leaf ratio (Huber value) are key determinants in modulating water transport and growth rates (Buckley *et al*. 2006; Pratt *et al*. 2017). Xylem anatomical traits, such as vessel area and density, as well as the proportion of parenchyma and fibres, provide insight into spatial usage related to resource allocation, hydraulic transport efficiency, and drought adaptations (Zanne *et al*. 2010; Gleason *et al*. 2012; Bittencourt *et al*. 2016), leading to ecological trade‐offs (Cosme *et al*. 2017), where species with higher hydraulic efficiency tend to have xylem that is more susceptible to embolism (Cosme *et al*. 2017; Avila *et al*. 2023). Although reported as a weak linear trade‐off (Gleason *et al*. 2015; Liu *et al*. 2021), recent studies indicate a convex relationship between efficiency and safety in water transport (Pereira *et al*. 2023).

In addition to water availability, soil conditions, such as the nutrient availability, also influence variations in plant functional traits, potentially explaining up to 77% of the variation observed in leaf traits, which highlights the importance of soil in modulating species' ecological strategies (Thomas *et al*. 2020; Joswig *et al*. 2022). Thus, studies seeking to understand the relationship between topographic position (edge and interior), nutrient availability, and plant functional responses provide a broader view of the mechanisms shaping the functioning of tree species in tropical forests. Considering the current scenario of intense fragmentation in the Amazon, a region with the highest absolute rate of forest destruction in the world (Gatti *et al*. 2021), understanding how species respond to these environmental changes is essential for predicting their future dynamics, assessing their resilience, and supporting more effective conservation strategies.

**Fig. 1:**
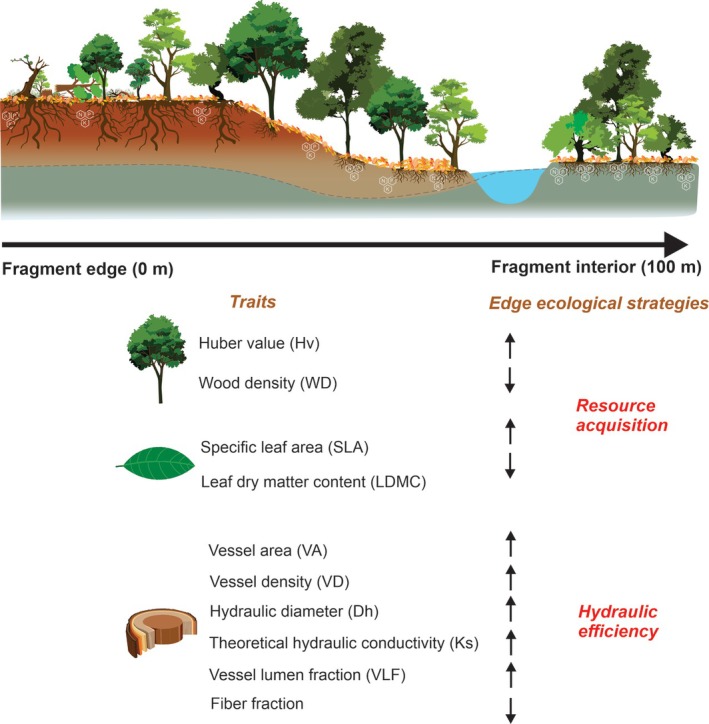
Diagram of the study hypotheses for the edge of fragments, highlighting the functional differences between species located at the edge and in the interior of forest fragments in the Eastern Amazon.

This study investigates the ecological strategies of plant species located at the edge and the interior of forest fragments in the Eastern Amazon. For this, we analysed leaf and anatomical traits of the abundant species, correlating them with the edaphic characteristics of the fragments. We propose two hypotheses: (i) pre‐existing trees in the interior of the fragments – established before fragmentation – will have more conservative and hydraulic safety traits, as evidenced by lower SLA, Hv, VA, VLF, Ks, and Dh, as well as higher fiber fraction, LDMC, and WD compared to trees that colonize the edges (mainly pioneer species), which display an acquisitive profile, prioritizing hydraulic efficiency; and (ii) the edaphic conditions – particularly higher nutrient content and water availability in the fragment interiors – will enable establishment of new species with increased water transport capacity and acquisitive strategies (Fig. 1).

## MATERIAL AND METHODS

### Study area

**Fig. 2:**
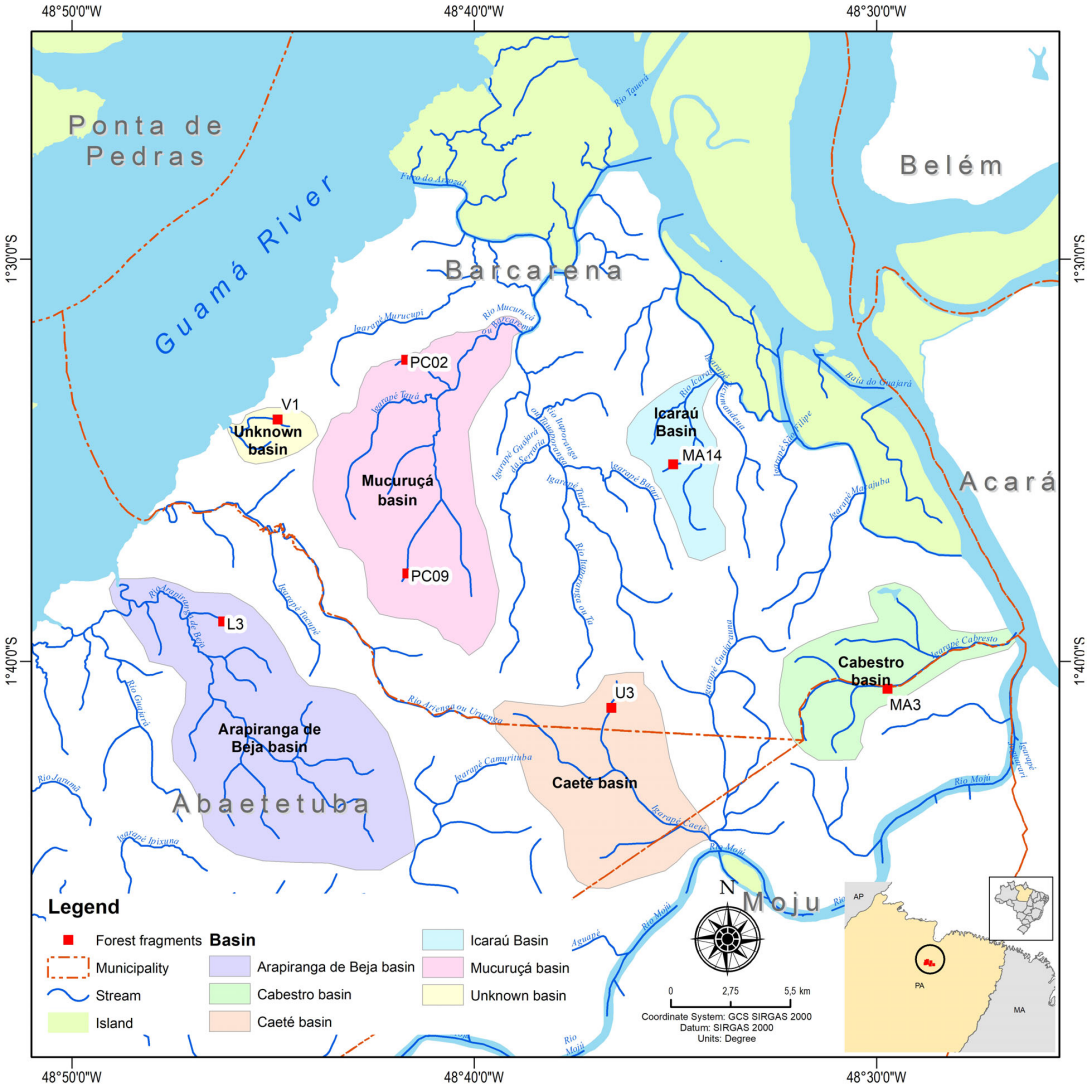
Forest fragments selected for the study of functional leaf and stem traits of tree species in the municipalities of Barcarena, Abaetetuba, and Moju, Pará, Brazil.

The study was carried out in seven forest fragments, distributed in three municipalities: Barcarena, Abaetetuba, and Moju, and six micro‐basins located in the state of Pará, in the Eastern Amazon (Fig. 2). The fragments exhibit topographic variation between the edge and the interior, with three fragments having higher variation between edge and interior (U3: 26.066 m; PC09: 11.714 m; MA3: 8.928 m), while other four fragments have lower variation (MA14: 2.303 m; V1: 2.276 m; PC02: 1.079 m and L3: 1.004 m). The edge is more elevated and in the interior of the studied fragments where there is a stream. The region has a tropical climate, classified as “Af” according to the Köppen‐Geiger classification (Alvares *et al*. 2013). The average annual temperature is 26.9°C (Piratoba *et al*. 2017), and the dominant vegetation is dense ombrophilous forest, with areas of floodplain forest along the Pará and Acará rivers (Souza & Lisboa 2005).

In each study fragment, we selected four to eight species and three individuals per species to monitor during the study, in total 45 records corresponding to 30 distinct species (Table S1). All collected specimens were identified by a taxonomist, and voucher numbers are provided in Table S1. The specimens were deposited in the Herbarium Professora Normélia Vasconcelos at UFPA (HF). The species were selected based on their abundance and importance to the Amazon flora. Among the collected species, 25 occurred at the edge of the fragments, 20 in the interior, and 5 species occur in more than one fragment. The data collection took place in February and March 2024.

### Sampling of functional traits

To analyse whether there are differences in plant strategies between edge and interior, and which traits are retained in forest fragments, we analysed 16 traits, including leaf morphological traits and wood morphological and anatomical traits. The means and standard deviations for all measured traits are provided in Supporting Information 2. For leaf analyzes, all leaves from the collected 60 cm terminal branch were used to determine total leaf area, while 10 leaves were selected for measuring specific functional traits, such as leaf area (LA), specific leaf area (SLA), and leaf dry matter content (LDMC), following the protocol proposed by Pérez‐Harguindeguy *et al*. (2013). In the laboratory, the leaves were rehydrated in water for 4 h, scanned, and their images analysed using R software to calculate leaf area (Maracahipes‐Santos *et al*. 2023). After rehydration, the leaves were weighed on a precision analytical balance (0.001 g) to obtain saturated fresh mass and subsequently dried in an oven at 65°C for 72 h to determine dry mass.

From these measurements, we calculated the specific leaf area (SLA, mm^2^ mg^−1^) as the ratio of leaf area to dry mass of leaves, and leaf dry matter content (LDMC, mg g^−1^) by dividing dry mass by saturated fresh mass. Huber values (mm^2^ cm^−2^) were calculated by dividing the sapwood area (measured with a calliper) by the branch leaf area.

At 60‐cm height, we collected two branch fragments ca. 5‐cm long to analyse wood density and anatomical traits. Wood density (g cm^−3^) was determined using Archimedes' principle. For this, a 5‐cm segment was submerged in water to measure the displaced volume, while dry mass was obtained after drying in an oven at 65°C for 72 h. Density was calculated by dividing dry mass by displaced volume.

For the histological sections, a 5‐cm branch segment was fixed in FAA solution (formalin:acetic acid:alcohol) for 2 days and then stored in 70% alcohol. Transverse sections with a thickness of 5 to 20 μm were made using a sliding microtome and cleared with sodium hypochlorite (NaClO) solution. The sections were stained with 1% Toluidine Blue and 1% Safranin, washed with distilled water, immersed in xylene for 5 min, and mounted on semi‐permanent slides with 50% glycerin. Images of the sections were captured using a microscope with an attached camera (Leica DM6 B, Germany) at 100× magnification, using a 10× objective lens. The field of view was standardized to the sapwood region closest to the bark, where vessels are actively growing. The images were processed using ImageJ software (v.1.8.0_345), employing the Threshold Analyse Particles tool for automatic cell counting. The analysed anatomical traits included vessel area (VA), vessel density (VD), hydraulic diameter (DH), theoretical specific conductivity (Ks), and vessel lumen fraction (VLF).

Vessel area (VA) was estimated for all vessels present in the image (Scholz *et al*. 2013). Vessel density (VD) was obtained from the frequency of all vessels (Scholz *et al*. 2013; Apgaua *et al*. 2015). Hydraulic diameter (DH) was estimated using the equation of Tyree and Zimmermann (2002)

$$\mathrm{DH}={\left(\frac{\sum D^{4}}{N}\right)}^{\frac{1}{4}}$$

where *D* represents diameter and *N* the number of vessels.

Theoretical specific hydraulic conductivity (Ks) was estimated using the Hagen‐Poiseuille equation

$$K_{s}=\pi \;\Sigma d^{4}/12{\text{8ηA}}_{\text{cross}-\text{section area}}$$

where ƞ is viscosity (1.002 × 10^−9^ MPa s^−1^) of water at 20°C (Tyree & Zimmermann 2002), D is diameter of the vessels, and A is area.

The vessel lumen fraction (VLF) was determined by summing all vessel area values and expressing this area as a percentage of total xylem area, using the equation of Avila *et al*. (2023):

$$\mathrm{VLF}=\frac{\sum A}{\text{Xylem area}}\times 100$$

To estimate the relative proportions of different cell types in the anatomical images, we defined a square with dimensions of 1140.18 × 1140.18 μm in ImageJ software. Within this square, we imposed a grid of 25 intersections in each structure, totaling 100 intersections. At each intersection point, we classified the cell types into three categories: (a) Vessel elements (including lumens and walls of vascular cells), (b) Axial parenchyma, and (c) fibers (comprising lumens and walls of fibrous cells) (Tng *et al*. 2018).

### Soil variables

Soil properties were evaluated in each fragment by collecting six soil samples at a depth of 0–20 cm, with three samples in the edge and three in the fragment interior. The levels of phosphorus (P), potassium (K), total nitrogen (N), soil pH, organic carbon (C), and granulometry (sand, clay, and silt) were estimated. The analyses were conducted by the Fullin Agronomic, Environmental, and Consulting Laboratory Ltd. in Linhares, Espírito Santo, Brazil. The methods used by the laboratory follow the protocol established by Embrapa (2009).

### Data analysis

To test the study's hypotheses, we selected the functional traits based on a Pearson correlation matrix (Figure S1). The selected traits were SLA, LDMC, Hv, WD, VLF, Ks, Dh, VA, and fiber fraction. The entire dataset underwent logarithmic transformation to meet the assumptions of normality and homoscedasticity.

To answer question (i) on which ecological strategies prevail at the edges and interior of forest fragments, we initially performed a principal components analysis (PCA) to assess arrangement of species based on functional traits. Next, we applied a PERMANOVA to test differences in functional composition between environments, and a PERMIDISP to evaluate dispersion of data within each group. Finally, we conducted a *t*‐test to compare trait means between edge and interior of the fragments.

To answer question (ii), on whether soils with higher nutrient content favour species with faster and more efficient functional strategies, a *t*‐test was first conducted to verify whether nutrient availability differs between the edge and the interior of fragments. Subsequently, multiple regression models were fitted for each of the nine selected traits. These models considered the interaction between edge/interior and edaphic variables (N, P, K, soil pH, organic C and clay content). To evaluate the quality of the fitted models, we used statistical criteria, such as AIC, and conducted residual diagnostics (e.g., Shapiro–Wilk test and residual plots) to ensure that model assumptions were met. All analyses were performed in R software.

## RESULTS

### Ecological strategies associated with the edge and interior of forest fragments

**Fig. 3:**
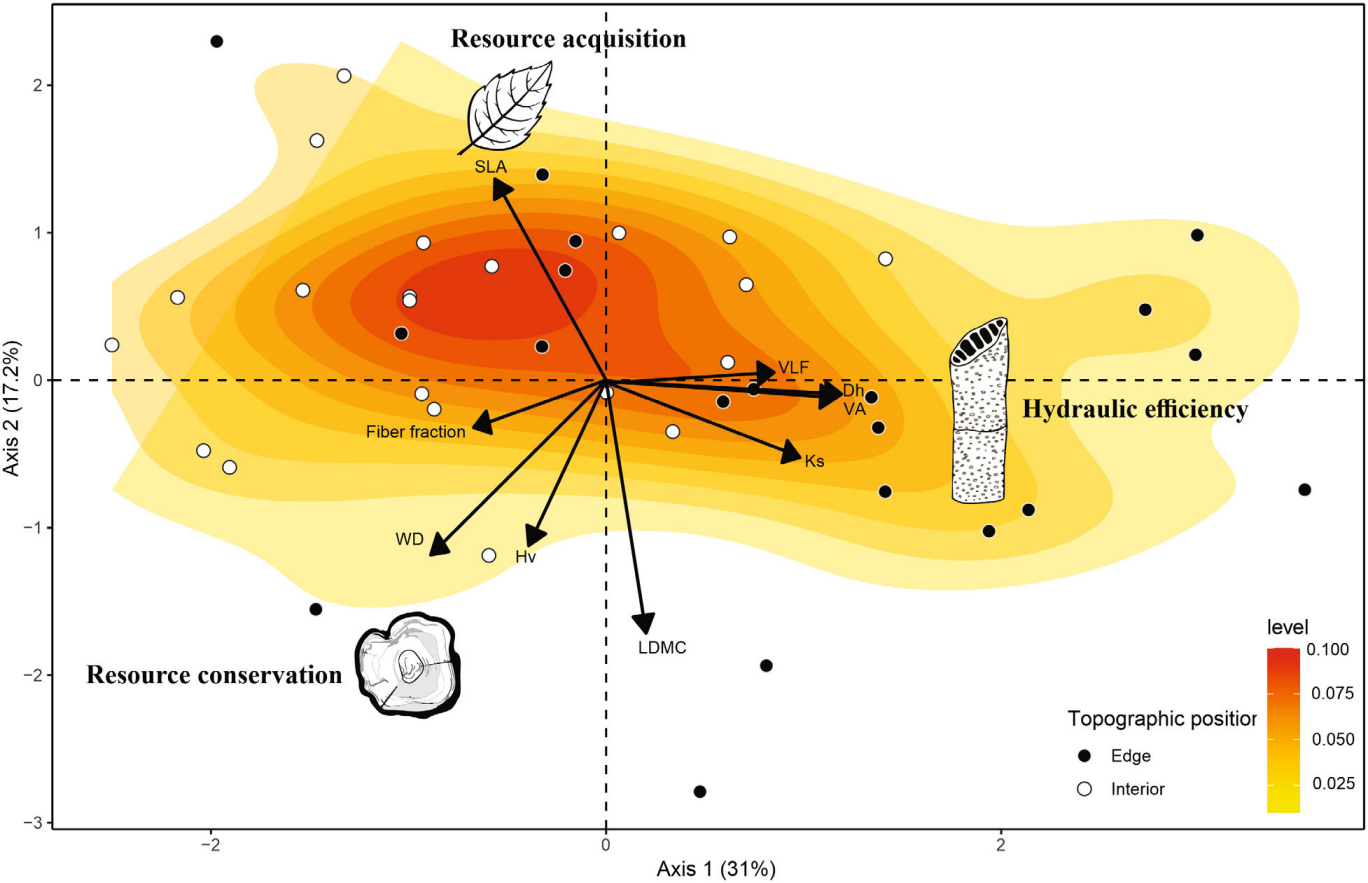
Principal components analysis performed with the functional traits (Dh, hydraulic diameter; Hv, Huber value; Ks, theoretical specific conductivity; LDMC, leaf dry matter content; SLA, specific leaf area; VA, vessel area; VLF, fiber fraction, vessel lumen fraction; WD, wood density) of species located at the edge and in the interior of forest fragments in the eastern Amazon. Black points represent species at the edge, while white points represent species in the fragment interior.

The results of PERMANOVA and PERMIDISP showed differences in species' trait values between the edge and the interior of fragments (PERMANOVA: Pseudo‐*F* = 5.15, *P* = 0.001; PERMIDISP: *F* = 5.174, *P* = 0.028). The first axis of the PCA represented 31% of total data variance, and from this it was possible to identify the separation of three groups (Fig. 3 and Table S2). The first group represented a strategy related to resource acquisition, with specific leaf area (SLA), associated with interior species. The second group represented species that invested in hydraulic efficiency, with vessel lumen fraction (VLF), theoretical specific conductivity (Ks), hydraulic diameter (Dh), and vessel area (VA), associated with edge species. The third group represented resource conservation, associated with traits such as fiber fraction, wood density (WD), Huber value (Hv), and leaf dry matter content (LDMC), associated with interior species. The separation of these groups also indicated an orthogonal axis, with one axis defined by the leaf traits and the other related to wood density and anatomical traits (Fig. 3).

**Fig. 4:**
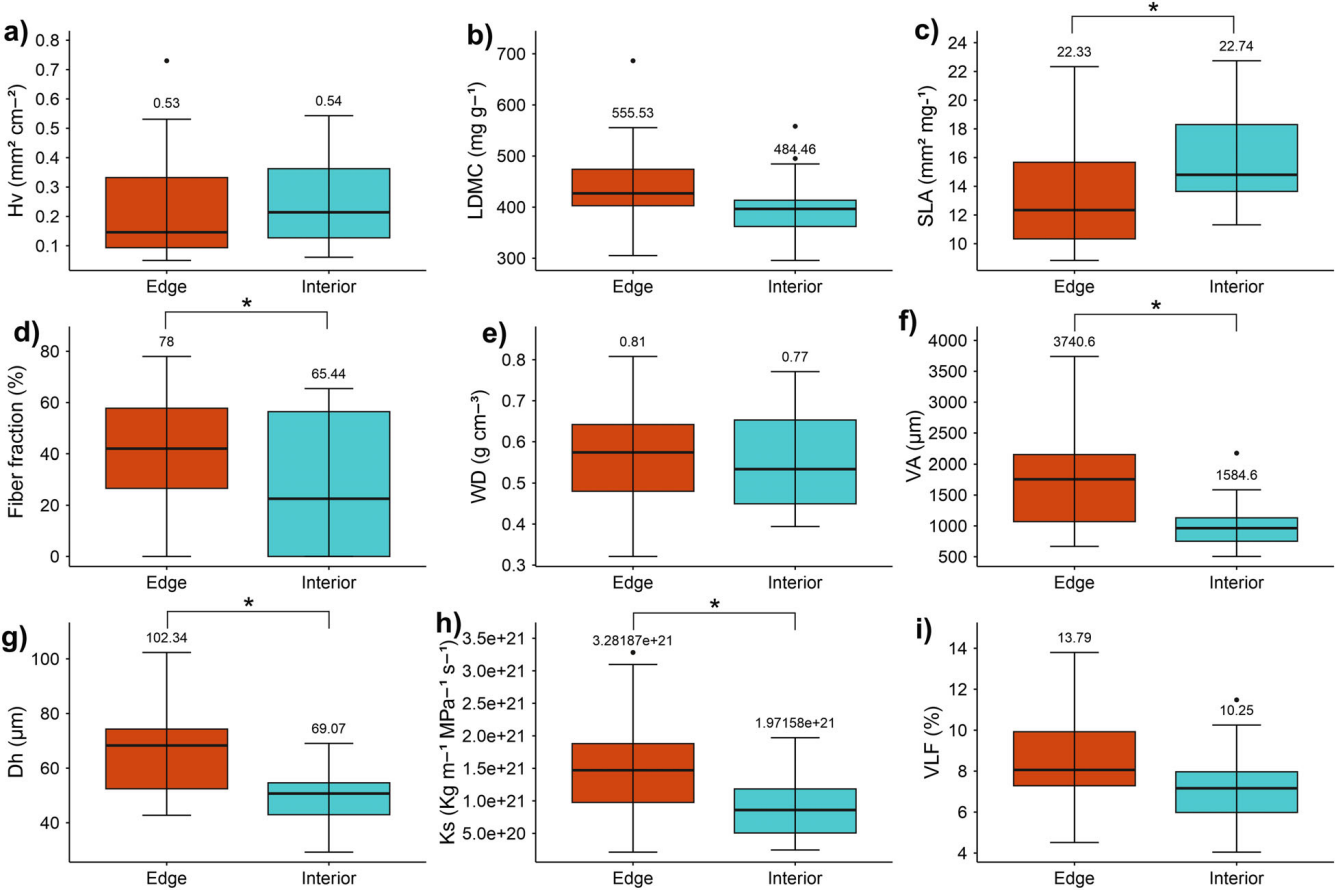
Results of *t*‐test for the functional traits: (a) Huber value (Hv), (b) leaf dry matter content (LDMC), (c) specific leaf area (SLA), (d) fiber fraction, (e) wood density (WD), (f) vessel area (VA), (g) hydraulic diameter (Dh), (h) theoretical specific conductivity (Ks) and (i) vessel lumen fraction (VLF) of species located at the edge and interior of forest fragments in the eastern Amazon. Asterisks indicate *P* < 0.05.

When comparing traits between edge and interior, the interior of the fragments had a higher SLA, while the edge of the fragments had higher values of Dh, VA, Ks, VLF, and fiber fraction. On the other hand, there were no significant differences for the other traits analysed, such as Hv, LDMC, and WD, between species in fragments, edge, or interior (Fig. 4 and Table S3).

### Effect of edaphic variables and topographic position on species functional traits in forest fragments

**Fig. 5:**
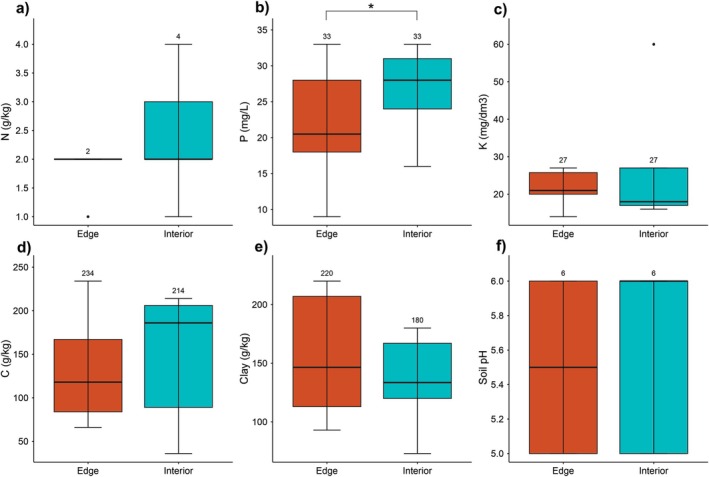
Results of *t‐*test comparing the edaphic variables: (a) nitrogen (N), (b) phosphorus (P), (c) potassium (K), (d) organic carbon (C), (e) clay, and (f) pH between edge and interior of forest fragments in the Eastern Amazon. Asterisks indicate *P* < 0.05.

When comparing edaphic variables per topographic position, only phosphorus (P) differed between edge and interior, with a higher concentration in the interior (Fig. 5). On the other hand, there were no significant differences for the other nutrients: nitrogen (N), potassium (K), organic carbon (C), clay content, and soil pH (Fig. 5 and Table S4).

We only observed significant relationships between some edaphic variables and functional traits for species in the fragment edge. LDMC and VA exhibited negative relations with nitrogen (LDMC: *P* = 0.023, *R* ^2^ adj = 0.413; VA: *P* = 0.011, *R* ^2^ adj = 0.554) and organic carbon (LDMC: *P* = 0.001, *R* ^2^ adj = 0.413; VA: *P* < 0.001, *R* ^2^ adj = 0.554), and positive relations with phosphorus (*P*; LDMC: *P* = 0.015, *R* ^2^ adj = 0.413; VA: *P* = 0.000, *R* ^2^ adj = 0.554) and potassium (LDMC: *P* = 0.000, *R* ^2^ adj = 0.413; VA: *P* = 0.000, *R* ^2^ adj = 0.554). Dh was positively correlated with phosphorus (*P* = 0.005), potassium (*P* = 0.006), and soil pH (*P* = 0.001), with an *R* ^2^ adj = 0.438. None of the analysed variables explained the variation in Hv, SLA, WD, Ks, VLF, or fiber fraction (Table S5).

## DISCUSSION

The ecological strategies of pre‐existing trees in the interior of the fragments, established before forest fragmentation, differ from those of trees that occur in the fragment edges in Eastern Amazonia. The species located at the forest edge exhibited strategies focused on hydraulic efficiency, with higher hydraulic conductivity and fiber fraction, while those in the fragment interior adopted different strategies, with some species showing a resource acquisition strategy, while others had a conservative strategy. We identified evidence of a complementary and decoupling relationship between investments in leaf and wood economic spectrum, defining the plant strategies, with axes of the wood economic spectrum and anatomical traits being orthogonal to the axis of variation in leaf traits. Additionally, edaphic conditions influenced traits resource allocation patterns of species only at the fragment edges.

### Ecological strategies associated with the edge and interior of forest fragments

Fragment edges contained species with traits indicative of higher hydraulic efficiency, including a higher vessel lumen fraction (VLF), theoretical specific conductivity (Ks), hydraulic diameter (Dh), and vessel area (VA). This pattern suggests that fragment edges, with higher abundance of generally pioneer species (Laurance *et al*. 2006b; Tabarelli *et al*. 2010), tend to invest in larger vessel areas with higher hydraulic conductivity. Pioneer species are more hydraulically efficient and can meet the increased water demand for photosynthesis; however, their xylem vessels may be more vulnerable to embolism formation (Markesteijn *et al*. 2011). Therefore, this investment in high hydraulic transport efficiency might be a risky strategy in environments subject to increased climatic variability, mainly considering the increase in drought events in the Amazon region.

In addition to investing in hydraulic efficiency, species at the edge had a higher fiber fraction compared to species in the fragment's interior, a strategy that highlights an adaptive response to adverse conditions of these areas. Fragment edges are characterized by a drier microclimate, with lower water availability and higher solar radiation exposure, causing plants to operate under more negative water potentials (Laurance *et al*. 2011; Nunes *et al*. 2023). This increases the risk of conduit collapse related to implosive forces in the xylem, requiring more resistant structures to compensate for negative pressures, such as a higher fiber fraction (Hacke *et al*. 2001; Hacke *et al*. 2005; Ziemińska *et al*. 2013; Bittencourt *et al*. 2016). The higher fiber fraction also provides mechanical support to withstand bending forces, such as winds, which are more frequent at fragment edges (Niklas *et al*. 2006).

The interior of the fragments had a diversity of ecological strategies, with some species possessing more acquisitive traits, while others had more conservative traits. This environment is composed of pre‐existing trees that were present before forest fragmentation and retain many characteristics of the original forest, as well as species that established themselves later, possibly new recruits or migrants from the edges through seed dispersal (Nunes *et al*. 2023). This coexistence suggests that while the older species may adopt more conservative strategies, the new species tend to follow more acquisitive traits, better taking advantage of available resources, such as light and nutrients.

Leaf and stem functional traits varied along distinct axes, with wood traits covarying orthogonally to leaf traits. This pattern suggests a possible decoupling between the economic spectrum of stems and leaves. Life history theory suggests that plant functional strategies are organized along a continuous axis, where species with low‐density wood have greater stem volume expansion, high hydraulic conductance, increased water supply to leaves, high photosynthetic rates, and, consequently, high growth rates (Baraloto *et al*. 2010). At the opposite end of this spectrum, species with denser wood tend to exhibit increased persistence and survival (Baraloto *et al*. 2010; Reich 2014). However, our results showed an orthogonal variation between leaf morphological traits versus wood density and anatomical traits, suggesting that coordination between leaf and stem spectra may not be universal. This observed decoupling may reflect different selective pressures acting on these trait sets, with environmental factors, such as water availability, soil nutrients, and topographic variation, independently influencing leaf and stem strategies (Díaz *et al*. 2016).

### Effect of edaphic variables and topographic position on functional traits of species in forest fragments

The edge of the fragments had lower phosphorus availability compared to the interior. This difference can be partially attributed to small‐scale topographic variations, as edges are generally located at slightly higher elevations (Oliveira *et al*. 2018; Mattos *et al*. 2023). In contrast, the interior tends to accumulate more leaf litter, which plays a crucial role in nutrient cycling and contributes to increased soil fertility. The increased input of organic matter in these areas enhances long‐term nutrient replenishment, especially of phosphorus, a limiting nutrient in tropical ecosystems (Ordoñez *et al*. 2009).

Soil variables emerged as important drivers of functional trait variation at forest fragment edges. However, contrary to our initial hypothesis, environments with lower nutrient availability were associated with shifts in functional traits. Vessel area (VA) and hydraulic diameter (Dh) had a positive correlation with soil pH and phosphorus (P), and a negative correlation with nitrogen (N). In more acidic soils, overall nutrient availability tends to decrease, as is the case for phosphorus, because more phosphate is adsorbed or precipitated, with Fe^3+^, Al^3+^, and Mn^2+^ ions released during soil acidification (Vitousek *et al*. 2010). Therefore, this positive correlation is not entirely clear, since lower nutrient availability is expected in more acidic soils. This result may indicate a possible physiological adjustment by species located at the edges of these fragments to maximize resources uptake (Lambers *et al*. 2006; Lambers *et al*. 2008).

Leaf dry matter content (LDMC) was influenced by the two most limiting nutrients for plants in tropical ecosystems (N and P) (Sullivan *et al*. 2019; Botelho *et al*. 2025), as well as by soil pH. The positive relationship with soil pH and the negative relationship with nitrogen are expected, since LDMC tends to increase as soil fertility decreases. In this context, plants invest in denser cellular structures and longer‐lived leaves, maximizing the retention of these essential limited resources for productivity (Wright *et al*. 2004). However, the positive relationship with phosphorus (P) is contrary to what would be expected according to the leaf economic spectrum (Wright *et al*. 2004). An important factor to consider is pH, as it affects the availability of phosphorus in the soil: reducing it. Thus, part of the phosphorus may not be available for absorption by plant species, which may explain the observed positive relationship (Rebelo *et al*. 2025).

Potassium was positively correlated with hydraulic diameter (Dh), vessel area (VA), and leaf dry matter content (LDMC). Potassium is considered a vital macronutrient and has significant roles in plants, such as osmoregulation, membrane potential regulation, sugar counter‐regulation, stress adaptation, and growth (Sanyal *et al*. 2020; Sardans & Peñuelas 2021). Potassium controls stomatal opening under drought conditions and helps plants acclimate to water stress (Aksu & Altay 2020; Pathak *et al*. 2020). Additionally, potassium can facilitate the construction of plant tissues (Sardans & Peñuelas 2021). Species growing in potassium‐rich soils may have increased durability and structural resilience, allowing them to better adapt to the adverse environmental conditions at the edges of fragments.

Organic carbon had a negative relationship with leaf dry matter content (LDMC) and vessel area (VA). Organic carbon is an important indicator of soil quality, influencing water retention capacity and availability of nutrients essential for plant growth (Lal 2004). Consequently, soils with higher organic carbon levels provide favourable conditions for species growth, which explains the lower LDMC observed in soils with elevated carbon content. On the other hand, the negative relationship with vessel area might suggest that soils rich in organic carbon favour species that prioritize hydraulic safety over efficiency, a relationship that is not yet fully understood.

### The role of functional traits in forest fragmentation dynamics

The analyses of species' functional traits in this study allowed us to identify the ecological strategies adopted at the edge and interior of forest fragments. In a scenario of increasing fragmentation in the Amazon, where the Amazon Basin faces the highest absolute rate of forest destruction globally (Gatti *et al*. 2021), understanding how species respond to this process is crucial for predicting their future dynamics. Forest fragmentation has various negative impacts, including increased tree mortality, changes in plant and animal species composition (Cushman 2006), greater susceptibility to forest fires (Cochrane *et al*. 2004), and increased access to the forest interior, thus intensifying hunting and logging (Peres 2001). Our study contributes to understanding of the mechanisms that regulate species survival, as evidenced by the different strategies adopted by plants in the interior and edge of the fragments, having contrasting strategies to cope with the resource availability. Thus, our results also provide support for conservation and forest management strategies, as well as assisting in the formulation of policies aimed at mitigating the impacts of fragmentation on Amazonian biodiversity.

## CONCLUSION

In conclusion, our study showed that ecological strategies in forest fragments are strongly influenced by edge effects. Pre‐existing trees in the interior had different strategies, varying from species adopting a resource acquisition strategy, to species with a conservative strategy, while pioneer species at the edge adopted a profile focused on hydraulic efficiency and a higher fiber fraction. We also found evidence of a decoupling between the leaf and wood economic spectra, indicating that these axes operate independently and are influenced by edaphic conditions, particularly at the edges of the fragments.

## AUTHOR CONTRIBUTIONS

TSS: writing – review and editing, methodology, investigation, formal analysis, data curation. RDP: review, methodology, investigation, data curation. LP: review, methodology, investigation, supervision. AB: review, methodology, investigation, data curation. LGB: methodology, investigation, data curation. TSM: review, project administration, methodology. RMC: review, data curation, botanical material identification. ESCG: data curation. GST: review, methodology, supervision, project planning.

## Supporting information


**Figure S1.** Pearson correlation matrix between functional traits of forest fragments in the Eastern Amazon.
**Table S1.** List of species collected for measurement of foliar and anatomical traits in forest fragments in the Eastern Amazon.
**Table S2.** Results of the PCA, showing the variable loadings for specific leaf area (SLA), leaf dry matter content (LDMC), Huber value (Hv), wood density (WD), fibre fraction, vessel lumen fraction (VLF), theoretical specific conductivity (Ks), hydraulic diameter (Dh), and vessel area (VA). The table also presents eigenvalues for the first two axes (Axis I and Axis II), and the percentage variance explained by each axis.
**Table S3.** Results of the *t*‐test showing degrees of freedom (DF), *t*‐values (T), and *P*‐values for the variables specific leaf area (SLA), leaf dry matter content (LDMC), Huber value (Hv), wood density (WD), fibre fraction, vessel lumen fraction (VLF), theoretical specific conductivity (Ks), hydraulic diameter (Dh), and vessel area (VA). Significant values are in bold.
**Table S4.** Results of *t*‐test showing degrees of freedom (DF), *t*‐values (T), and p‐values for the edaphic soil variables (C, organic carbon; K, potassium; N, nitrogen; P, phosphorus; pH, and clay content). Significant values are in bold.
**Table S5.** Relationship between functional traits (Dh, hydraulic diameter; Hv, huber value; Ks, theoretical specific conductivity; LDMC, leaf dry matter content; SLA, specific leaf area; VA, vessel area; VLF, fibre fraction, vessel lumen fraction; WD, wood density), and soil variables (C, organic carbon; K, potassium; N, nitrogen; P, phosphorus; pH, and clay content) and topographic position (edge and interior) adjusted using linear regression models. Significant variables (*P* < 0.05) are highlighted in bold.

Data S1.
