# Supplementary material for: Edge effect influences the ecological strategies of plant communities in tropical forest fragments
Source: Plant Biol (Stuttg). 2025 Nov 11;28(2):509–19. doi: 10.1111/plb.70137 (PMC12884026; doi:10.1111/plb.70137)
Supplement: Supplementary file 1 — Figure S1. Pearson correlation matrix between functional traits of forest fragments in the Eastern Amazon. Table S1. List of species collected for measurement of foliar and anatomical traits in forest fragments in the Eastern Amazon. Table S2. Results of the PCA, showing the variable loadings for specific leaf area (SLA), leaf dry matter content (LDMC), Huber value (Hv), wood density (WD), fibre fraction, vessel lumen fraction (VLF), theoretical specific conductivity (Ks), hydraulic diameter (Dh), and vessel area (VA). The table also presents eigenvalues for the first two axes (Axis I and Axis II), and the percentage variance explained by each axis. Table S3. Results of the t‐test showing degrees of freedom (DF), t‐values (T), and P‐values for the variables specific leaf area (SLA), leaf dry matter content (LDMC), Huber value (Hv), wood density (WD), fibre fraction, vessel lumen fraction (VLF), theoretical specific conductivity (Ks), hydraulic diameter (Dh), and vessel area (VA). Significant values are in bold. Table S4. Results of t‐test showing degrees of freedom (DF), t‐values (T), and p‐values for the edaphic soil variables (C, organic carbon; K, potassium; N, nitrogen; P, phosphorus; pH, and clay content). Significant values are in bold. Table S5. Relationship between functional traits (Dh, hydraulic diameter; Hv, huber value; Ks, theoretical specific conductivity; LDMC, leaf dry matter content; SLA, specific leaf area; VA, vessel area; VLF, fibre fraction, vessel lumen fraction; WD, wood density), and soil variables (C, organic carbon; K, potassium; N, nitrogen; P, phosphorus; pH, and clay content) and topographic position (edge and interior) adjusted using linear regression models. Significant variables (P < 0.05) are highlighted in bold. [file PLB-28-509-s002.docx]

**Edge effect influences the ecological strategies of plant communities in tropical forest fragments**

^1^Tailane Silva Sousa ^*^, ^1^Renan Domingues Pacheco, ^2^Luciano Pereira, ^3^Anthony Barbosa, ^4^Luane Gabriela Botelho, ^5^Thaisa Sala Michelan, ^5^Roberta MacedoCerqueira, ^3^Ely Simone Cajueiro Gurgel, ^5^Grazielle Sales Teodoro ^*^.

**Table S1.** List of species collected for the measurement of foliar and anatomical traits in forest fragments in the Eastern Amazon.

| **Fragment** | **Voucher** | **Position** | **Species name** | |
| --- | --- | --- | --- | --- |
| U3 | 8060 | Interior | | *Miconia affinis DC.* |
| U3 | 8061 | Interior | | *Theobroma grandiflorum* (Willd. ex Spreng.) K.Schum. |
| U3 | 8062 | Interior | | *Endopleura uchi* (Huber) Cuatrec. |
| U3 | 8063 | Interior | | *Vantanea parviflora* Lam. |
| U3 | 8064 | Edge | | *Calliandra surinamensis* Benth. |
| U3 | 8065 | Edge | | *Vismia guianensis* (Aubl.) Choisy. |
| U3 | 8066 | Edge | | *Inga alba* (Sw.) Willd. |
| MA3 | 8067 | Edge | | *Vismia guianensis* (Aubl.) Choisy. |
| MA3 | 8068 | Edge | | *Theobroma grandiflorum* (Willd. ex Spreng.) K.Schum. |
| MA3 | 8069 | Edge | | *Inga heterophylla* Willd. |
| MA3 | 8070 | Edge | | *Banara guianensis* Aubl. |
| MA3 | 8071 | Interior | | *Myrcia sylvatica* (G.Mey.) DC. |
| MA3 | 8072 | Interior | | *Protium aracouchini* (Aubl.) Marchand. |
| MA3 | 8073 | Interior | | *Guatteria schomburgkiana* Mart. |
| MA3 | 8074 | Interior | | *Iryanthera juruensis* Warb. |
| PC02 | 8075 | Edge | | *Vismia guianensis* (Aubl.) Choisy. |
| PC02 | 8076 | Edge | | *Humiria balsamifera* (Aubl.) A.St.-Hil. |
| PC02 | 8077 | Edge | | *Calliandra surinamensis* Benth. |
| PC02 | 8078 | Interior | | *Miconia affinis DC.* |
| PC02 | 8079 | Interior | | *Calliandra surinamensis* Benth. |
| PC02 | 8080 | Interior | | *Myrciaria tenella* (DC.) O.Berg. |
| V1 | 8081 | Edge | | *Miconia cuspidata* Naudin. |
| V1 | 8082 | Edge | | *Deguelia spruceana* (Benth.) A.M.G.Azevedo & R.A.Camargo. |
| V1 | 8083 | Edge | | *Guarea macrophylla* Vahl. |
| V1 | 8084 | Interior | | *Guarea macrophylla* Vahl. |
| V1 | 8085 | Interior | | *Miconia cuspidata* Naudin. |
| V1 | 8086 | Interior | | *Aparisthmium cordatum* (A.Juss.) Baill. |
| L3 | 8087 | Edge | | *Vismia guianensis* (Aubl.) Choisy. |
| L3 | 8088 | Edge | | *Inga edulis* Mart. |
| L3 | 8089 | Edge | | *Xylopia frutescens* Aubl. |
| L3 | 8090 | Interior | | *Theobroma grandiflorum* (Willd. ex Spreng.) K.Schum. |
| L3 | 8091 | Interior | | *Simarouba amara* Aubl. |
| L3 | 8092 | Interior | | *Macrosamanea pubiramea* (Steud.) Barneby & J.W.Grimes var. *pubiramea*. |
| PC09 | 8093 | Edge | | *Carapa guianensis* Aubl. |
| PC09 | 8094 | Edge | | *Vismia guianensis* (Aubl.) Choisy. |
| PC09 | 8095 | Edge | | *Xylopia ferruginea* (Hook.f. & Thomson) Baill. |
| PC09 | 8096 | Interior | | *Theobroma grandiflorum* (Willd. ex Spreng.) K.Schum. |
| PC09 | 8097 | Interior | | *Caraipa grandifolia* Mart. |
| MA14 | 8098 | Edge | | *Miconia cuspidata* Naudin. |
| MA14 | 8099 | Edge | | *Vismia guianensis* (Aubl.) Choisy. |
| MA14 | 8100 | Edge | | *Inga thibaudiana* DC*.* |
| MA14 | 8101 | Edge | | *Discophora guianensis* Miers. |
| MA14 | 8102 | Interior | | *Calliandra surinamensis* Benth. |
| MA14 | 8103 | Interior | | *Siparuna guianensis* Aubl. |
| MA14 | 8104 | Interior | | *Endlicheria anomala* (Nees) Mez. |


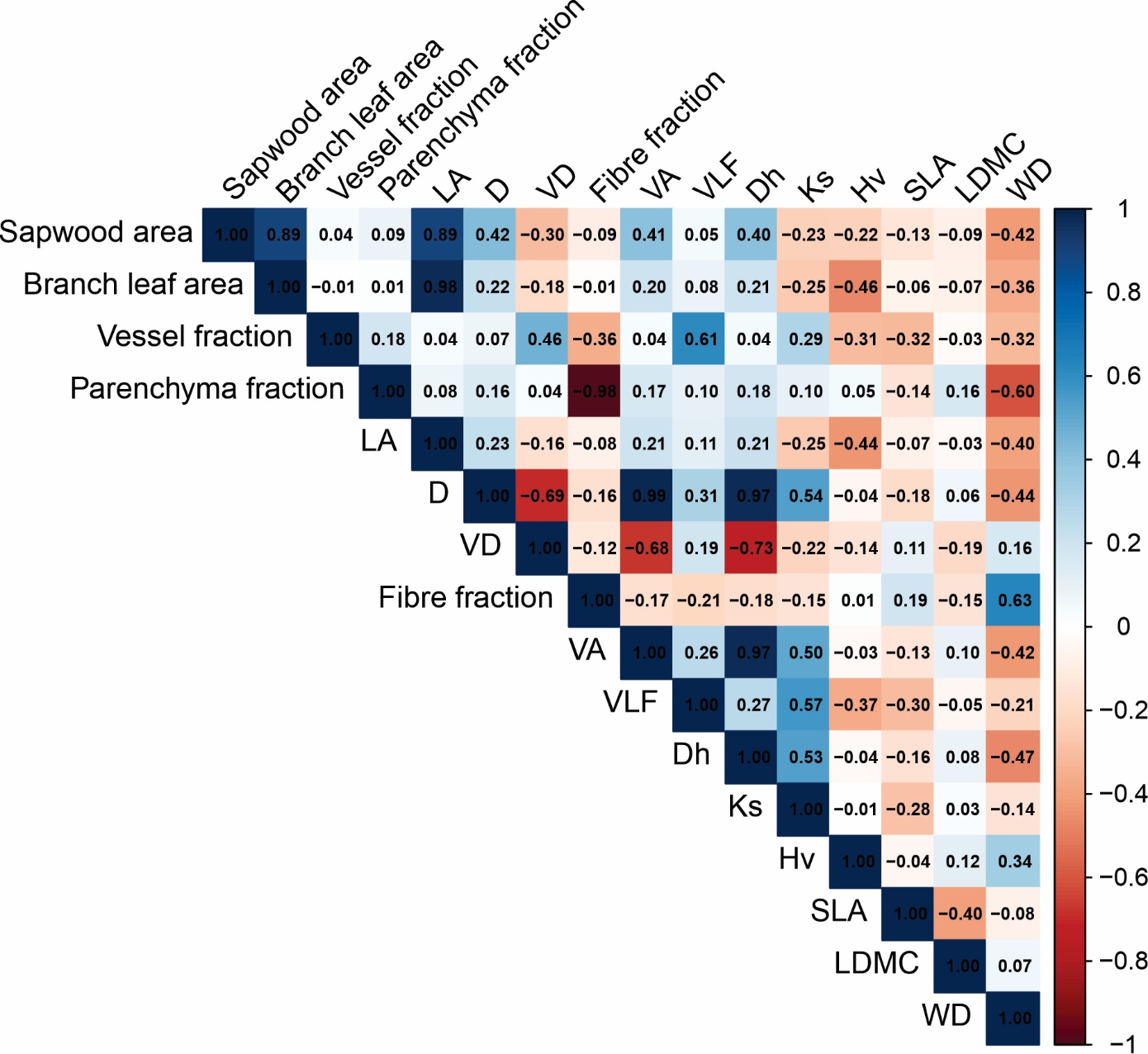


**Figure S1.** Pearson correlation matrix between functional traits of forest fragments in the Eastern Amazon.

**Table S2.** Results of the Principal Component Analysis (PCA), showing the variable loadings for specific leaf area (SLA), leaf dry matter content (LDMC), Huber value (Hv), wood density (WD), fiber fraction, vessel lumen fraction (VLF), theoretical specific conductivity (Ks), hydraulic diameter (Dh), and vessel area (VA). The table also presents the eigenvalues for the first two axes (Axis I and Axis II) and the percentage of variance explained by each axis.

| **Traits** | **Axis I** | **Axis II** |
| --- | --- | --- |
| VLF | 0.598 | 0.033 |
| Dh | 0.857 | -0.041 |
| Ks | 0.705 | -0.236 |
| Hv | -0.224 | -0.543 |
| SLA | -0.339 | 0.666 |
| LDMC | 0.133 | -0.653 |
| WD | -0.630 | -0.549 |
| VA | 0.836 | -0.052 |
| Fibre fraction | -0.478 | -0.149 |
| Eigenvalue | 3.100 | 1.551 |
| Broken Stick | 2.828 | 1.828 |
| % explanation | 34.447 | 17.243 |

**Table S3.** Results of the t-test showing degrees of freedom (DF), t values (T), and p-values for the variables specific leaf area (SLA), leaf dry matter content (LDMC), Huber value (Hv), wood density (WD), fiber fraction, vessel lumen fraction (VLF), theoretical specific conductivity (Ks), hydraulic diameter (Dh), and vessel area (VA). Significant values are shown in bold.

| **Traits** | **DF** | **T** | | **p-Value** |
| --- | --- | --- | --- | --- |
| VLF | 39 | 2.330 | **0.025** | |
| Dh | 39 | 2.199 | **0.032** | |
| Ks | 39 | 2.215 | **0.032** | |
| Hv | 39 | -0.651 | 0.518 | |
| SLA | 39 | -2.369 | 0.022 | |
| LDMC | 39 | 1.369 | 0.178 | |
| WD | 39 | 0.157 | 0.875 | |
| VA | 39 | 2.199 | **0.033** | |

**Table S4.** Results of the t-test showing degrees of freedom (DF), t values (T), and p-values for the edaphic soil variables (nitrogen – N, phosphorus – P, potassium – K, organic carbon – C, pH, and clay content). Significant values are shown in bold.

| **Traits** | **DF** | **T** | | **p-Value** |
| --- | --- | --- | --- | --- |
| N | 42 | -1.293 | 0.202 | |
| P | 42 | -2.400 | **0.020** | |
| K | 42 | -1.175 | 0.246 | |
| C | 42 | -0.809 | 0.422 | |
| Clay | 42 | 1.395 | 0.170 | |
| Soil pH | 42 | -0.295 | 0.769 | |

**Table S5.** Relationship between functional traits (specific leaf area – SLA, leaf dry matter content – LDMC, Huber value – Hv, wood density – WD, fiber fraction, vessel lumen fraction – VLF, theoretical specific conductivity – Ks, hydraulic diameter – Dh and vessel area – VA) and soil variables (nitrogen- N, phosphorus - P, potassium - K, organic carbon (C), pH, and clay content) and topographic position (edge and interior) adjusted using linear regression models. Significant variables (p < 0.05) are highlighted in bold.

| **Hv (AIC=-57.878; R² adj= 0.171)** | | | | | **SLA (AIC=-0.337; R² adj= 0.229)** | | | | | |
| --- | --- | --- | --- | --- | --- | --- | --- | --- | --- | --- |
|  | Estimates | Std.  Error | F | p-value | Estimates | Std.  Error | | F | p-value |  |
| **N:Edge** | 0.270 | 0.722 | 0.374 | 0.711 | -0.107 | 1.360 | -0.079 | | 0.937 |  |
| **N:Interior** | -0.671 | 0.609 | -1.102 | 0.279 | 1.151 | 1.187 | 0.970 | | 0.340 |  |
| **P:Edge** | -0.184 | 0.205 | -0.896 | 0.377 | 0.180 | 0.401 | 0.449 | | 0.656 |  |
| **P:Interior** | 0.314 | 0.785 | 0.400 | 0.691 | -1.472 | 1.532 | -0.961 | | 0.344 |  |
| **K:Edge** | -0.573 | 0.754 | -0.759 | 0.453 | -0.226 | 1.387 | -0.163 | | 0.871 |  |
| **K:Interior** | 0.008 | 0.224 | 0.040 | 0.968 | -0.403 | 0.445 | -0.906 | | 0.372 |  |
| **pH:Edge** | -0.832 | 0.851 | -0.978 | 0.336 | -2.167 | 1.638 | -1.323 | | 0.195 |  |
| **pH:Interior** | -2.947 | 1.570 | -1.876 | 0.070 | 3.172 | 2.830 | 1.121 | | 0.271 |  |
| **Clay:Edge** | 0.166 | 0.248 | 0.670 | 0.508 | -0.348 | 0.451 | -0.771 | | 0.446 |  |
| **Clay:Interior** | 1.083 | 0.693 | 1.562 | 0.129 | -1.790 | 1.305 | -1.372 | | 0.180 |  |
| **C:Edge** | 0.452 | 0.505 | 0.895 | 0.378 | -0.078 | 0.932 | -0.084 | | 0.933 |  |
| **C:Interior** | -0.121 | 0.166 | -0.727 | 0.473 | 0.263 | 0.317 | 0.828 | | 0.414 |  |
| **LDMC (AIC=-50.166; R² adj= 0.413)** | | | | | **WD (AIC=-89.805; R² adj= 0.136)** | | | | | |
| **N:Edge** | -1.887 | 0.788 | -2.395 | **0.023** | -0.393 | 0.484 | -0.811 | | 0.424 |  |
| **N:Interior** | -0.128 | 0.658 | -0.196 | 0.846 | 0.832 | 0.485 | 1.715 | | 0.097 |  |
| **P:Edge** | 0.576 | 0.224 | 2.574 | **0.015** | -0.062 | 0.136 | -0.458 | | 0.650 |  |
| **P:Interior** | 0.632 | 0.869 | 0.727 | 0.473 | 0.370 | 0.533 | 0.694 | | 0.493 |  |
| **K:Edge** | 2.497 | 0.791 | 3.154 | **0.003** | 0.124 | 0.501 | 0.249 | | 0.805 |  |
| **K:Interior** | 0.327 | 0.249 | 1.312 | 0.200 | 0.089 | 0.150 | 0.596 | | 0.556 |  |
| **pH:Edge** | 3.954 | 0.879 | 4.496 | **0.000** | 0.000 | 0.566 | 0.010 | | 0.991 |  |
| **pH:Interior** | -0.100 | 1.701 | -0.059 | 0.953 | 0.404 | 0.958 | 0.422 | | 0.676 |  |
| **Clay:Edge** | -0.303 | 0.265 | -1.142 | 0.263 | -0.076 | 0.165 | -0.465 | | 0.645 |  |
| **Clay:Interior** | 0.340 | 0.748 | 0.455 | 0.652 | -0.800 | 0.488 | -1.639 | | 0.112 |  |
| **C:Edge** | -1.867 | 0.547 | -3.409 | **0.001** | -0.016 | 0.3335 | -0.048 | | 0.961 |  |
| **C:Interior** | -0.015 | 0.181 | -0.085 | 0.933 | 0.002 | 0.106 | 0.024 | | 0.981 |  |
| **VA (AIC=38.259; R² adj= 0.554)** | | | | | **Dh (AIC=-5.304; R² adj=0.438)** | | | | | |
| **N:Edge** | -5.647 | 2.100 | -2.100 | **0.011** | -2.237 | 1.255 | -1.782 | | 0.084 |  |
| **N:Interior** | -1.337 | 1.886 | -0.709 | 0.483 | -1.106 | 1.127 | -0.982 | | 0.334 |  |
| **P:Edge** | 2.674 | 0.634 | 4.218 | **0.000** | 1.140 | 0.379 | 3.009 | | **0.005** |  |
| **P:Interior** | 4.385 | 2.572 | 1.704 | 0.098 | 1.957 | 1.537 | 1.273 | | 0.212 |  |
| **K:Edge** | 8.694 | 2.172 | 4.003 | **0.000** | 3.769 | 1.298 | 2.903 | | **0.006** |  |
| **K:Interior** | 0.977 | 0.752 | 1.299 | 0.204 | 0.437 | 0.448 | 0.976 | | 0.337 |  |
| **pH:Edge** | 11.821 | 2.732 | 4.326 | **0.000** | 5.612 | 1.633 | 3.437 | | **0.001** |  |
| **pH:Interior** | -6.945 | 4.534 | -1.532 | 0.136 | -4.259 | 2.710 | -1.572 | | 0.126 |  |
| **Clay:Edge** | 1.046 | 0.727 | -1.439 | 0.160 | -0.402 | 0.434 | -0.926 | | 0.361 |  |
| **Clay:Interior** | 3.525 | 2.067 | 1.705 | 0.098 | 2.402 | 1.235 | 1.944 | | 0.061 |  |
| **C:Edge** | -6.164 | 1.475 | -4.177 | **0.000** | -2.704 | 0.310 | -1.482 | | 0.148 |  |
| **C:Interior** | -0.871 | 0.519 | -1.678 | 0.104 | -0.460 | 0.310 | -1.482 | | 0.148 |  |
| **Ks (AIC=87.881; R² adj=-0.150)** | | | | | **VLF (AIC=-7.196; R² adj= 0.268)** | | | | | |
| **N:Edge** | -29.892 | 47.204 | -0.633 | 0.531 | 0.308 | 1.266 | 0.244 | | 0.809 |  |
| **N:Interior** | 2.326 | 3.872 | 0.601 | 0.552 | 0.631 | 1.207 | 0.523 | | 0.605 |  |
| **P:Edge** | -1.108 | 3.308 | -0.335 | 0.740 | 0.068 | 0.366 | 0.188 | | 0.852 |  |
| **P:Interior** | -1.286 | 8.667 | -0.148 | 0.883 | 0.692 | 1.398 | 0.495 | | 0.625 |  |
| **K:Edge** | 20.687 | 30.281 | 0.683 | 0.500 | 0.370 | 1.251 | 0.296 | | 0.769 |  |
| **K:Interior** | -0.299 | 2.569 | -0.117 | 0.908 | 0.315 | 0.407 | 0.775 | | 0.445 |  |
| **pH:Edge** | -21.693 | 50.550 | -0.429 | 0.671 | 1.913 | 1.472 | 1.300 | | 0.205 |  |
| **pH:Interior** | 7.592 | 10.515 | 0.722 | 0.476 | 2.155 | 2.767 | 0.779 | | 0.443 |  |
| **Clay:Edge** | 2.425 | 3.746 | 0.647 | 0.522 | 0.432 | 0.414 | 1.044 | | 0.306 |  |
| **Clay:Interior** | -2.704 | 4.052 | -0.667 | 0.510 | -0.392 | 1.408 | -0.278 | | 0.783 |  |
| **C:Edge** | 0.105 | 4.726 | 0.022 | 0.982 | -0.262 | 0.849 | -0.309 | | 0.760 |  |
| **C:Interior** | 0.630 | 1.462 | 0.431 | 0.669 | -0.036 | 0.302 | -0.120 | | 0.906 |  |
| **Fibre fraction (AIC=149.423; R² adj= 0.170)** | | | | |  | | | | | |
| **N:Edge** | 0.734 | 8.019 | 0.092 | 0.928 |  |  |  | |  |  |
| **N:Interior** | -7.873 | 7.280 | -1.081 | 0.288 |  |  |  | |  |  |
| **P:Edge** | -0.776 | 2.509 | -0.310 | 0.759 |  |  |  | |  |  |
| **P:Interior** | -8.017 | 9.150 | -0.876 | 0.388 |  |  |  | |  |  |
| **K:Edge** | -1.764 | 8.339 | -0.212 | 0.834 |  |  |  | |  |  |
| **K:Interior** | -0.510 | 2.663 | -0.192 | 0.849 |  |  |  | |  |  |
| **pH:Edge** | -8.812 | 9.683 | -0.910 | 0.370 |  |  |  | |  |  |
| **pH:Interior** | -0.066 | 17.155 | -0.004 | 0.997 |  |  |  | |  |  |
| **Clay:Edge** | -1.295 | 2.688 | -0.482 | 0.634 |  |  |  | |  |  |
| **Clay:Interior** | 3.114 | 8.158 | 0.382 | 0.705 |  |  |  | |  |  |
| **C:Edge** | 0.724 | 5.638 | 0.129 | 0.899 |  |  |  | |  |  |
| **C:Interior** | -1.107 | 1.915 | -0.578 | 0.568 |  |  |  | |  |  |
